# Identification of an endogenous glutamatergic transmitter system controlling excitability and conductivity of atrial cardiomyocytes

**DOI:** 10.1038/s41422-021-00499-5

**Published:** 2021-04-06

**Authors:** Duanyang Xie, Ke Xiong, Xuling Su, Guanghua Wang, Qiang Ji, Qicheng Zou, Lingling Wang, Yi Liu, Dandan Liang, Jinfeng Xue, Luxin Wang, Xueting Gao, Xingdong Gu, Hongyu Liu, Xiaoyu He, Li Li, Jian Yang, Youming Lu, Luying Peng, Yi-Han Chen

**Affiliations:** 1 Department of Cardiology, East Hospital, Tongji University School of Medicine, Shanghai, 200120 China; 2 Key Laboratory of Arrhythmias of the Ministry of Education of China, Tongji University School of Medicine, Shanghai, 200120 China; 3 Institute of Medical Genetics, Tongji University, Shanghai, 200092 China; 4 Department of Cardiovascular Surgery, Zhongshan Hospital, Fudan University, Shanghai, 200032 China; 5 Jinzhou Medical University, Jinzhou, Liaoning 121000 China; 6 Department of Regenerative Medicine, Tongji University School of Medicine, Shanghai, 200092 China; 7 Reproductive Medicine Center, Tongji Hospital, Tongji University School of Medicine, Shanghai, 200065 China; 8 Department of Pathology and Pathophysiology, Tongji University School of Medicine, Shanghai, 200092 China; 9 Institute for Brain Research, Wuhan Center of Brain Science, School of Basic Medicine and Tongji Medical College, Huazhong University of Science and Technology, Wuhan, Hubei 430030 China

**Keywords:** Cell biology, Molecular biology

## Abstract

As an excitatory transmitter system, the glutamatergic transmitter system controls excitability and conductivity of neurons. Since both cardiomyocytes and neurons are excitable cells, we hypothesized that cardiomyocytes may also be regulated by a similar system. Here, we have demonstrated that atrial cardiomyocytes have an intrinsic glutamatergic transmitter system, which regulates the generation and propagation of action potentials. First, there are abundant vesicles containing glutamate beneath the plasma membrane of rat atrial cardiomyocytes. Second, rat atrial cardiomyocytes express key elements of the glutamatergic transmitter system, such as the glutamate metabolic enzyme, ionotropic glutamate receptors (iGluRs), and glutamate transporters. Third, iGluR agonists evoke iGluR-gated currents and decrease the threshold of electrical excitability in rat atrial cardiomyocytes. Fourth, iGluR antagonists strikingly attenuate the conduction velocity of electrical impulses in rat atrial myocardium both in vitro and in vivo. Knockdown of *GRIA3* or *GRIN1*, two highly expressed iGluR subtypes in atria, drastically decreased the excitatory firing rate and slowed down the electrical conduction velocity in cultured human induced pluripotent stem cell (iPSC)-derived atrial cardiomyocyte monolayers. Finally, iGluR antagonists effectively prevent and terminate atrial fibrillation in a rat isolated heart model. In addition, the key elements of the glutamatergic transmitter system are also present and show electrophysiological functions in human atrial cardiomyocytes. In conclusion, our data reveal an intrinsic glutamatergic transmitter system directly modulating excitability and conductivity of atrial cardiomyocytes through controlling iGluR-gated currents. Manipulation of this system may open potential new avenues for therapeutic intervention of cardiac arrhythmias.

## Introduction

Glutamate is an excitatory neurotransmitter that mediates excitatory synaptic transmission in the central nervous system (CNS).^1^ Activity-dependent release of glutamate neurotransmitter from presynaptic terminals evokes excitatory postsynaptic currents and causes postsynaptic neuronal depolarization, resulting in the delivery of electrical excitation between neurons.^2,3^ It is known that extracellular glutamate activates both ionotropic glutamate receptors (iGluRs) and metabotropic glutamate receptors (mGluRs).^4^ iGluRs are ligand-gated ion channels with selective permeability to Na^+^, K^+^, and Ca^2+^, and can be subclassified into *N*-methyl-D-aspartate (NMDA) receptors, α-amino-3-hydroxy-5 methylisoxazole-4-propionate (AMPA) receptors, and kainite receptors based on selective agonists: NMDA, AMPA, and kainite, which can generate iGluR-gated currents to induce depolarization of membrane potential.^5^ mGluRs are G protein-coupled receptors that initiate intracellular second messenger cascades when activated by glutamate.^6^

Both cardiomyocytes and neurons are excitable cells that have the ability to generate action potentials in response to external stimuli.^7^ Since neurotransmitters can trigger action potentials (electrical excitations) in neurons, we hypothesize that they also have the potential to trigger action potentials in cardiomyocytes. Actually, previous studies have documented that some specific glutamate receptors are expressed in cardiomyocytes,^8–10^ suggesting that glutamate has the potential to regulate the excitability of cardiomyocytes. The ventricular myocardium has a unique electrical excitation conduction system, i.e., the His–Purkinje system, which controls both the sequential ventricular activation and the ventricular systolic synchrony.^11,12^ However, whether the atrial myocardium contains a similar conduction system has yet to be studied.^13^ Given that the atria do not have similar well-defined anatomic conduction system as the ventricles, we hypothesized that certain transmitters such as glutamate might play a compensatory role in the conduction of electrical excitation in the atrial myocardium.

In this study, we identified the key elements of glutamatergic transmitter system in rat atrial cardiomyocytes. The pulsatile administration of iGluR agonists in atrial cardiomyocytes induced iGluR-gated currents that facilitated the occurrence of action potentials. Intervention of the glutamatergic transmitter system not only attenuated the excitability and conductivity of these cells, but also suppressed the occurrence and development of atrial fibrillation (AF). The above findings were partially verified in human induced pluripotent stem cell (iPSC)-derived atrial cardiomyocytes, human atrial myocardial tissues, and human atrial cardiomyocytes. Taken together, we demonstrated that the glutamatergic transmitter system participates in controlling the excitability and conductivity of atrial cardiomyocytes.

## Results

### Vesicles containing glutamate located beneath the plasma membrane of the atrial cardiomyocytes

**Fig. 1:**
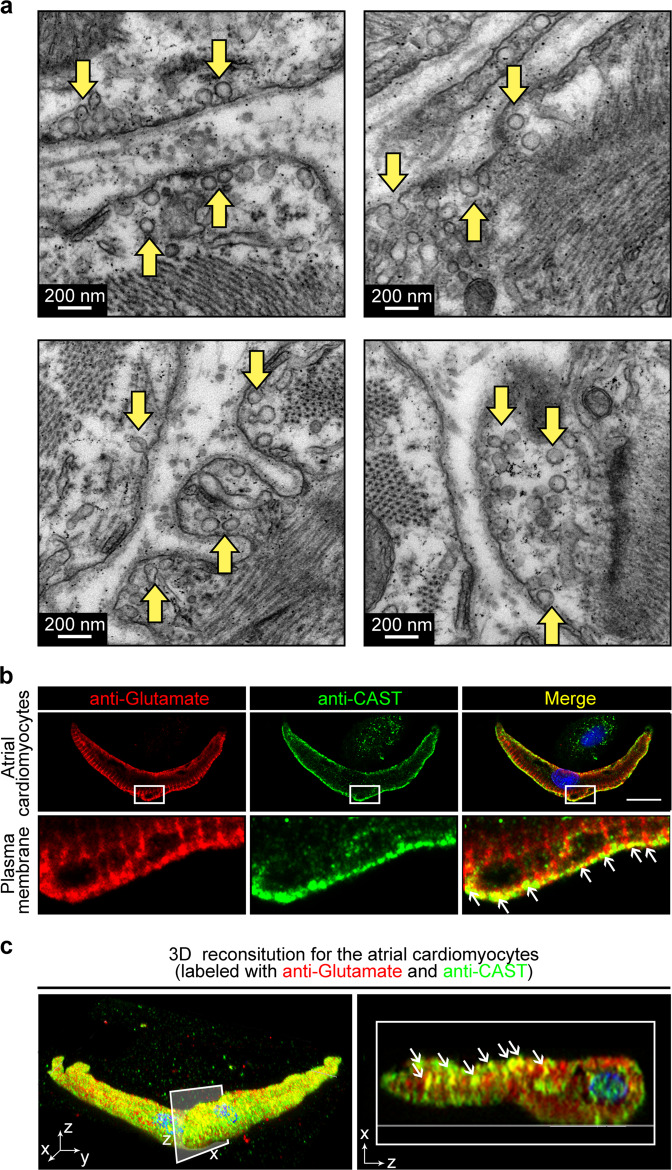
Observation of the vesicles containing glutamate in the rat atrial cardiomyocytes. **a** Representative TEM images showing vesicles beneath the plasma membrane of atrial cardiomyocytes (yellow arrows). Scale bar, 200 nm. **b** The immunofluorescence confocal images showing glutamate (red) colocalized with the secretory vesicles marker, CAST (green) in atrial cardiomyocytes. Scale bar, 10 μm. **c** 3D reconstruction from the *z*-stack confocal image showing colocalization of glutamate and CAST in the atrial cardiomyocyte. Left, 3D view of the whole cell labeled with glutamate antibody (red) and CAST antibody (green). Right, 2D section view of the *x-z* coordinates showing the colocalization (white arrows) of glutamate (red) and CAST (green) in the atrial cardiomyocyte.

In search of potential transmitter system in atrial cardiomyocytes, we employed the transmission electron microscopy (TEM) to examine the subcellular structure of atrial cardiomyocytes. Surprisingly, we observed numerous vesicles locating beneath the plasma membrane where two atrial cardiomyocytes connect (Fig. 1a). Glutamate is the primary excitatory neurotransmitter in the mammalian CNS.^14^ Hence, we labeled the atrial cardiomyocytes with a general anti-glutamate antibody and an antibody against CAST, a releasable vesicle marker. By confocal microscopy, we examined the spatial relationship of glutamate and the vesicles, which revealed the colocalization (yellow) of glutamate (red) and CAST (green) in a majority of vesicles beneath the plasma membrane. This result suggested that vesicles containing glutamate are present in atrial cardiomyocytes (Fig. 1b). To further validate the spatial colocalization of glutamate and the vesicles in atrial cardiomyocytes, we performed 3D construction for the *z*-stack immunofluorescence image of the atrial cardiomyocytes. As shown in Fig. 1c and Supplementary information, Video S1, the overall observation of the intact atrial cardiomyocytes labeled with the glutamate and CAST indicated that a large number of the vesicles containing glutamate exist next to the surface membrane of the atrial cardiomyocytes.

### Atrial cardiomyocytes express key elements of glutamatergic transmitter system

**Fig. 2:**
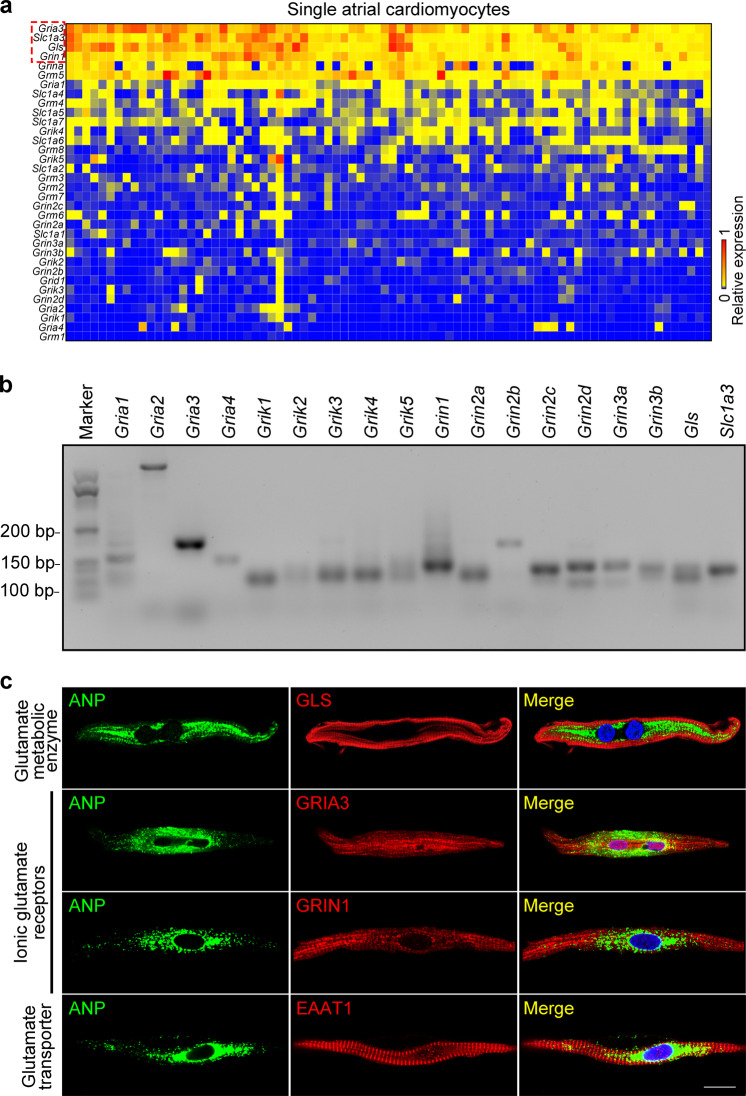
Rat atrial cardiomyocytes express core elements of the glutamatergic transmitter system. **a** Heat map showing expression of 34 assayed genes in single atrial cardiomyocytes. Rows are the samples of single atrial cardiomyocytes; columns are the genes. Color denotes the relative expression levels of the genes. **b** Representative agarose gel image of the semi-qPCR products demonstrats the expression of the glutamatergic transmitter system genes in atrial cardiomyocytes. **c** Immunofluorescent localization of GLS, GRIA3, GRIN1 and EAAT1 in ANP-expressing rat atrial cardiomyocytes. Scale bar, 25 μm.

After identifying the vesicles containing glutamate in atrial cardiomyocytes, we next analyzed the transcripts of the major components of the glutamatergic transmitter system in rat atrial cardiomyocytes by single-cell quantitative PCR (qPCR) and semi-qPCR. As shown in Fig. 2a, b, we found that *Gls* (encoding the glutaminase (GLS)), *Slc1a3* (encoding the excitatory amino acid transporter 1, EAAT1) and the two iGluR-coding genes, *Gria3* (encoding the APMP iGluR) and *Grin1* (encoding the NMDA iGluR) were highly expressed in rat atrial cardiomyocytes. In contrast, the expression of other iGluRs (such as *Gria1-2*, *Grin2-3,* and G*rik1-5*) was relatively low in atrial cardiomyocytes, indicating that GRIA3 and GRIN1 might be the main iGluRs on atrial cardiomyocytes. Subsequently, by using immunofluorescence and confocal imaging analyses, we confirmed the protein expression of the highly expressed components of glutamatergic transmitter system in rat atrial cardiomyocytes. As shown in Fig. 2c, GLS, iGluRs (GRIA3 and GRIN1), and the glutamate transporter (EAAT1) were all detected in rat cardiomyocytes labeled by atrial natriuretic peptide (ANP), an atrial-specific marker. Moreover, the results also suggested that GRIA3 and GRIN1 preferentially located on the membrane of the atrial cardiomyocytes, implying their potential functional role in regulating the transmembrane ionic currents of the atrial cardiomyocytes.

### Glutamate elicited iGluR-dependent transient inward current in rat atrial cardiomyocytes

**Fig. 3:**
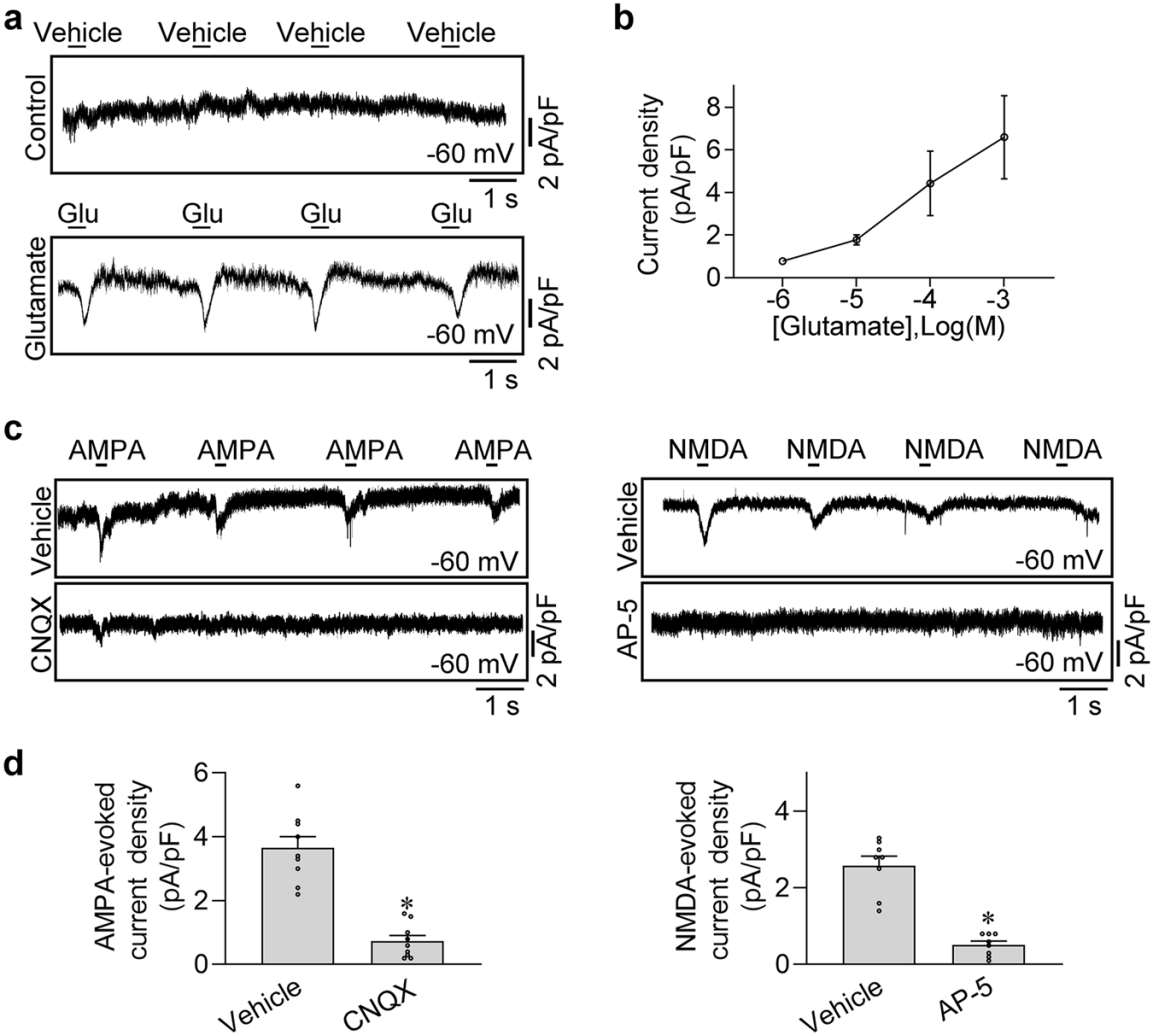
Identification of iGluRs-gated currents in the rat atrial cardiomyocytes. **a** Representative patch-clamp recordings of whole-cell currents elicited by glutamate in rat atrial cardiomyocytes. The holding potential was set at −60 mV. **b** Pooled data of the glutamate-elicited currents with different concentrations of glutamate. *n* = 9. **c** The AMPA or NMDA iGluR-gated currents in atrial cardiomyocytes were blocked by local application of CNQX or AP-5 in the perfusion solution at the holding potential of −60 mV. **d** Pooled data of the AMPA or NMDA iGluR-gated current densities from **c**. *P < 0.05, calculated by Student’s *t*-test, *n* = 9 per group.

Considering the high expression of GRIA3 and GRIN1 in atrial cardiomyocytes, we subsequently set to analyze the electrophysiological function of these two iGluRs in isolated rat atrial cardiomyocytes with specific agonists. We detected currents induced by the most common iGluR agonist, glutamate. The cells were seeded on the chamber in a Mg^2+^-free Tyrode solution with continuous perfusion. After setting the holding potential of the atrial cardiomyocytes to –60 mV, we sprayed glutamate solution onto the surface of the cells (5 ms, 30 psi) and recorded the currents in voltage-clamp mode. As shown in Fig. 3a, b, a transient inward current was elicited rapidly by the glutamate (1 mM), but not by the regular Tyrode solution in control. We then applied two other iGluR agonists, AMPA and NMDA, which can specifically activate the AMPA iGluR and the NMDA iGluR, respectively, to examine the effects of specific iGluR-mediated ionic currents in atrial cardiomyocytes. Notably, as shown in Fig. 3c, d, similar to the glutamate, a transient inward current was evoked by either 1 mM AMPA or 1 mM NMDA under the clamp voltage of –60 mV, which can be eliminated either by CNQX (the blocker of the AMPA iGluR) or by AP-5 (the blocker of the NMDA iGluR), respectively. These results strongly suggested that the iGluR-mediated currents can be elicited in rat atrial cardiomyocytes, which might play a role in triggering the action potential of atrial cardiomyocytes.

### iGluRs modified the excitatory plasticity in the atrial cardiomyocytes

**Fig. 4:**
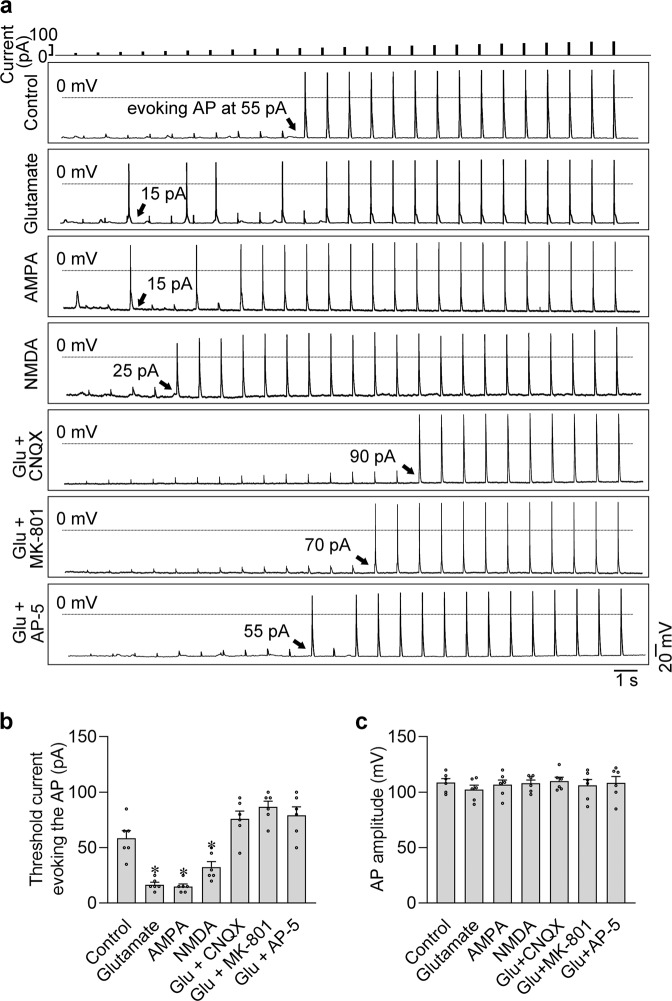
Activation of iGluRs excited the isolated rat atrial cardiomyocytes. **a** Action potentials were evoked by the increasing injection currents from 5 pA to 125 pA by 5 pA step. The cells were separately treated with iGluR agonist puffs (1 mM glutamate, 1 mM AMPA or 1 mM NMDA, respectively), or the iGluR antagonists (100 μM CNQX, MK-801 or AP-5, respectively), which were delivered through the perfusion solution. Black arrows indicate the minimum threshold current evoking the action potential. **b**, **c** Pooled data of the minimum threshold current evoking action potential and the amplitude of the action potentials from **a**. *P < 0.05, calculated by Student’s *t*-test, *n* = 6 per group.

To test the effects of the two iGluRs-mediated currents in exciting the atrial cardiomyocytes, we coupled the stimulation of iGluR agonist puff and a series of increasing current injections (from 5 pA to 125 pA by 5 pA step) to determine the minimum threshold current evoking the action potential. As shown in Fig. 4a, the application of glutamate, AMPA, or NMDA statistically lowered the minimum stimulation currents evoking the action potentials, increasing the excitability of the isolated atrial cardiomyocytes. Importantly, application with the iGluR antagonists, including 100 μM CNQX, MK-801, and AP-5, effectively eliminated the exciting effects of glutamate on atrial cardiomyocytes, further confirming the role of iGluRs in regulating the excitability of atrial cardiomyocytes (Fig. 4a, b). Interestingly, neither the agonists nor the antagonists changed the amplitude of the action potential, indicating that iGluRs mainly mediate the initial phase of the electrical excitation in atrial cardiomyocytes, which determines the subsequent action potential generation, but not the intensity of the electrical excitation (Fig. 4c).

### Inhibition of iGluRs attenuated the atrial electrical conduction velocity both in vivo and ex vivo

**Fig. 5:**
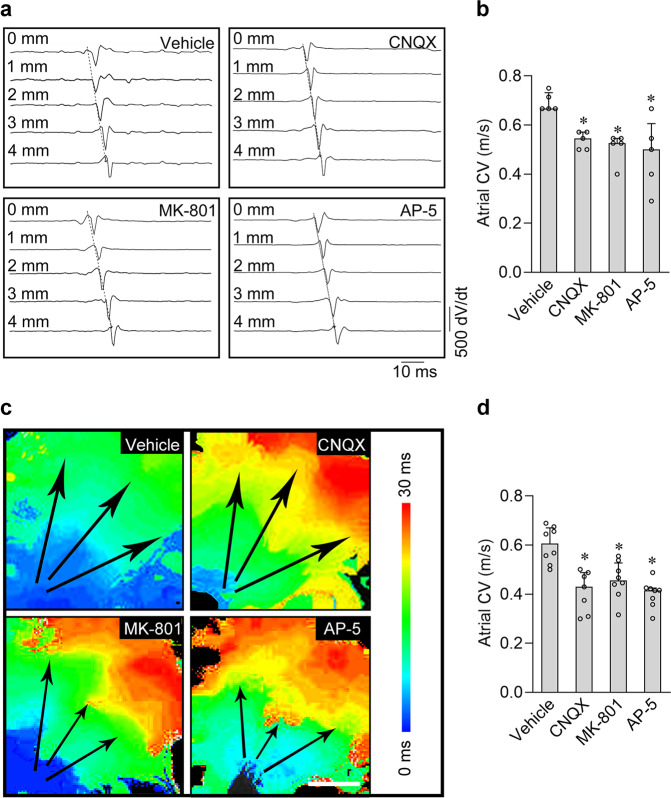
Inhibition of iGluRs attenuated electrical conduction velocity in rat atrial myocardium. **a** Representative right atrial ECG recordings (shown as the derivative of the electric potential as dV/dt) in anesthetic rats. The iGluR antagonists (CNQX, MK-801, and AP-5) respectively slow the electrical conduction velocity. **b** Pooled data of electrical conduction velocity from **a**. *P < 0.05, calculated by Student’s *t*-test or Kolmogorov–Smirnov test, *n* = 4−5 per group. **c** Typical conduction heatmap of isolated rat atria perfused with 100 μM CNQX, MK-801 or AP-5, respectively. The black arrows indicate the conduction direction of the action potential. Scale bar, 4 mm. **d** Pooled data of electrical conduction velocity from **c**. *P < 0.05, calculated by Student’s *t*-test, *n* = 7−8 per group.

Since the iGluRs are the major excitatory elements that directly control the electrical signal conduction between neurons, we speculated that blocking the iGluRs might interrupt the normal electric conduction in atria. To this end, we investigated the effects of three iGluR antagonists (CNQX, MK-801, or AP-5) on electric conduction of adult rat atria in vivo with the intra-atrial electrophysiology catheter or in isolated hearts by optical mapping (for ex vivo). As shown in Fig. 5a, b, we found that injection of 5 mg/kg iGluR antagonists (CNQX, MK-801, or AP-5) via intra-atrial electrophysiology catheter dramatically decreased the atrial conduction velocity of the anesthetic rats in 5 min. Similar results were also obtained in the isolated heart, which excludes the influence of peripheral autonomic nerves. We loaded the voltage-sensitive dye, Rh237 (30 µM) to the langendorff-perfused heart for 5 min, and collected the fluorescence signals in atria by a high-speed camera with 1000 frame per second. Subsequently, we measured the fluorescence signals and calculated the conduction velocity of the heart treated with drug vehicle (DMSO) and the 100 μM iGluR antagonists (CNQX, MK-801, or AP-5), respectively. As shown in Fig. 5c, d, compared with the data in vehicle group, all three antagonists reduced the atrial electrical conduction velocity in 5 min, indicating that iGluR antagonists inhibit the atrial conduction velocity in a rapid and direct working mode.

### Knockdown of *GRIA3* and *GRIN1* decreased the excitability and conductivity of human iPSC-derived atrial cardiomyocytes

**Fig. 6:**
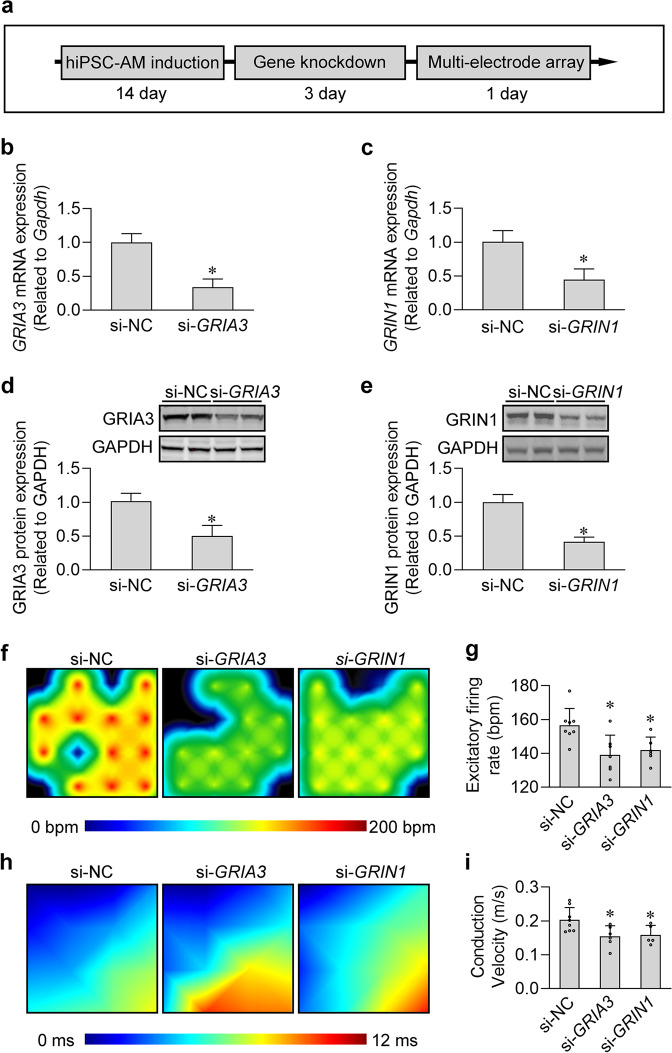
Knockdown of iGluRs decreased the excitability and conductivity in the cultured human iPSC-derived atrial cardiomyocyte monolayers. **a** Experimental protocol for the MEA analysis in cultured human iPSC-derived atrial cardiomyocyte monolayers. **b**, **c** Knockdown efficiency of *GRIA3* (**b**) and *GRIN1* (**c**) in the human iPSC-derived atrial cardiomyocytes at mRNA level. *P < 0.05, calculated by Student’s *t*-test, *n* = 3. **d**, **e** Knockdown efficiency of *GRIA3* (**d**) and *GRIN1* (**e**) in human iPSC-derived atrial cardiomyocytes at protein level. *P < 0.05, calculated by Student’s *t*-test, *n* = 3. **f** Typical heatmap of the excitatory firing rate in the cultured atrial cardiomyocyte monolayers with *GRIA3* or *GRIN1* knockdown. **g** Pooled data of firing rate from **f**. *P < 0.05, calculated by Student’s *t*-test, *n* = 5−7 per group. **h** Typical conduction heatmap of cultured atrial cardiomyocyte monolayers with *GRIA3* or *GRIN1* knockdown. **i** Pooled data of conduction velocity from **h**. *P < 0.05, calculated by Student’s *t*-test, *n* = 5−8 per group.

To circumvent any off-target effects that the iGluR agonists and antagonists may have in our electrophysiological assays, we next detected the effects of *GRIA3* and *GRIN1* knockdown on the excitability and conductivity of human iPSC-derived atrial cardiomyocyte monolayers by multiple microelectrode array (MEA) (Fig. 6a). We first determined the efficiency of gene knockdown by qPCR and western blotting. Results showed that *GRIA3* and *GRIN1* expression levels were reduced by more than 50% at both mRNA and protein levels in human iPSC-derived atrial cardiomyocytes (Fig. 6b–e). Next, by MEA, we found that knockdown of *GRIA3* and *GRIN1* statistically decreased the spontaneous firing rate and electrical conduction velocity of the atrial cardiomyocyte monolayers (Fig. 6f–i), implying that they are critical components of the glutamatergic transmitter system in regulating the excitability and conductivity of human atrial cardiomyocytes.

### Inhibition of iGluRs prevented and terminated AF

**Fig. 7:**
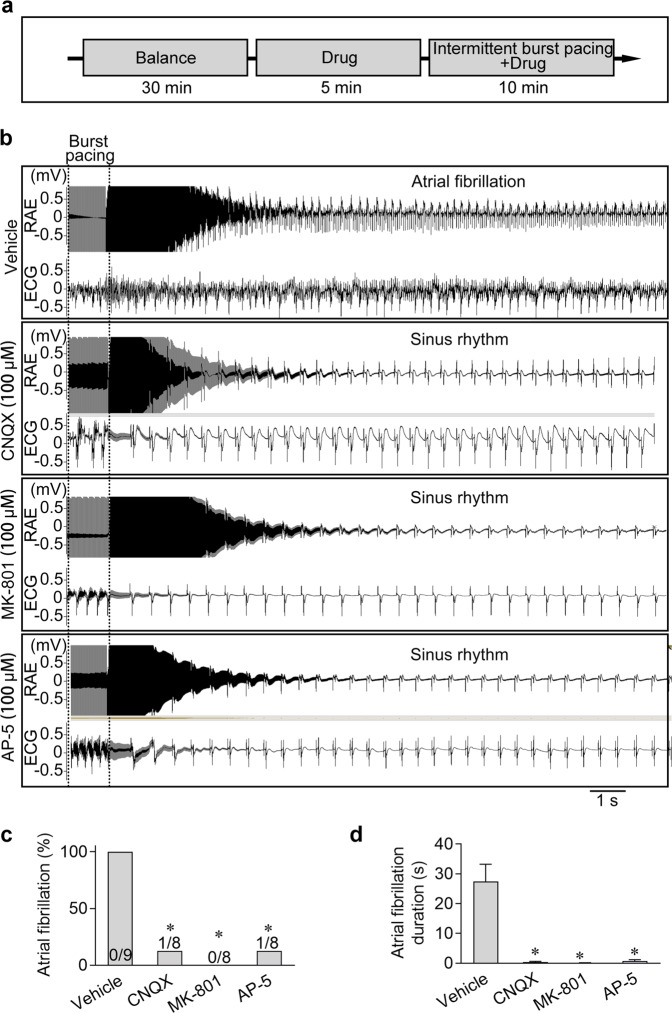
Blockage of iGluRs prevented AF. **a** Experimental design for analyzing the effects of the iGluR antagonists on AF. **b** Representative ECG and right atrial electrograms (RAE) in the heart showing that an atrial 40 Hz pacing protocol successfully induced AF in the vehicle control. However, pre-treatment with the iGluR antagonists, including CNQX, MK-801 or AP-5, prevented the occurrence of the AF under the same burst pacing stimulation. **c**, **d** Pooled data of the incidence and duration of AF induced by the 40 Hz burst pacing protocol in atria with or without the pre-treatment of the iGluR antagonists. *P < 0.05, calculated by *χ*^*2*^ test, *n* = 8−9.

**Fig. 8:**
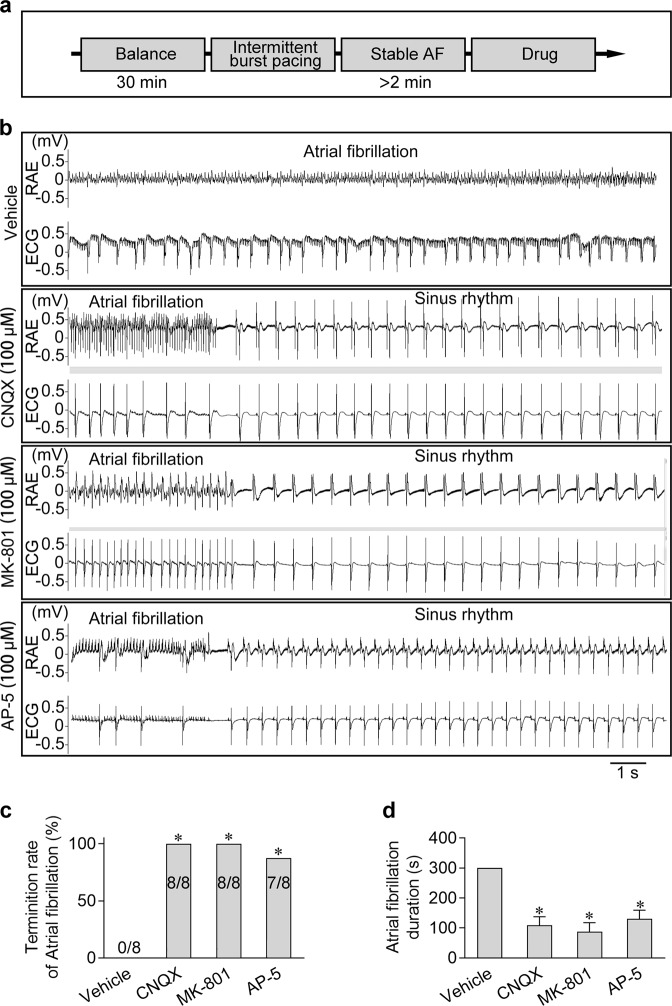
Blockage of iGluRs terminated AF. **a** Experimental design for analyzing the effects of the iGluR antagonists on treating AF. **b** Representative ECG and RAE in the heart showing that AF was terminated by the application of CNQX, MK-801, or AP-5 but not the vehicle. **c**, **d** Pooled data of the incidence and duration of the burst pacing-induced AF with or without treatment of the iGluR antagonists. *P < 0.05, calculated by *χ*^*2*^ test, *n* = 8−9.

In general, glutamate excitotoxicity induced by excessive stimulation or overactivation in neurons could cause multiple neuronal diseases,^15^ and iGluRs have been regarded as therapeutic targets for many glutamate excitotoxicity-related diseases.^16^ Abnormal excitability and conductivity of cardiomyocytes can also cause arrhythmias, among which AF is the most common one. It would be intriguing to understand the role of atrial glutamatergic transmitter system in the occurrence and progression of AF. We therefore applied iGluR blockers on the AF model. First, we tested the effects of iGluR antagonist pre-treatment on preventing AF (Fig. 7a). All three iGluR blockers (CNQX, MK-801, or AP-5) could dramatically decrease the induction rate and the duration of AF, implying that iGluR blockage significantly prevented AF occurrence (Fig. 7b–d). Next, to experimentally treat AF, we established a sustained AF model in vitro with decreased conventional perfusion (from 6 mL/min to 3 mL/min) and intermittent 40 Hz burst pacing. When AF occurred and lasted for over 2 min, the heart was immediately perfused with either CNQX, MK801, or AP-5 (Fig. 8a). In most cases, AF could be terminated within 2 min after iGluR antagonist perfusion, and the AF duration was dramatically shortened (Fig. 8b–d). These data suggested that the blockage of iGluRs can prevent the occurrence and suppress the progression of AF, indicating that the iGluRs may act as potential therapeutic targets for the prevention and treatment of AF.

### Identification of functional intrinsic glutamatergic transmitter system in human atrial cardiomyocytes

**Fig. 9:**
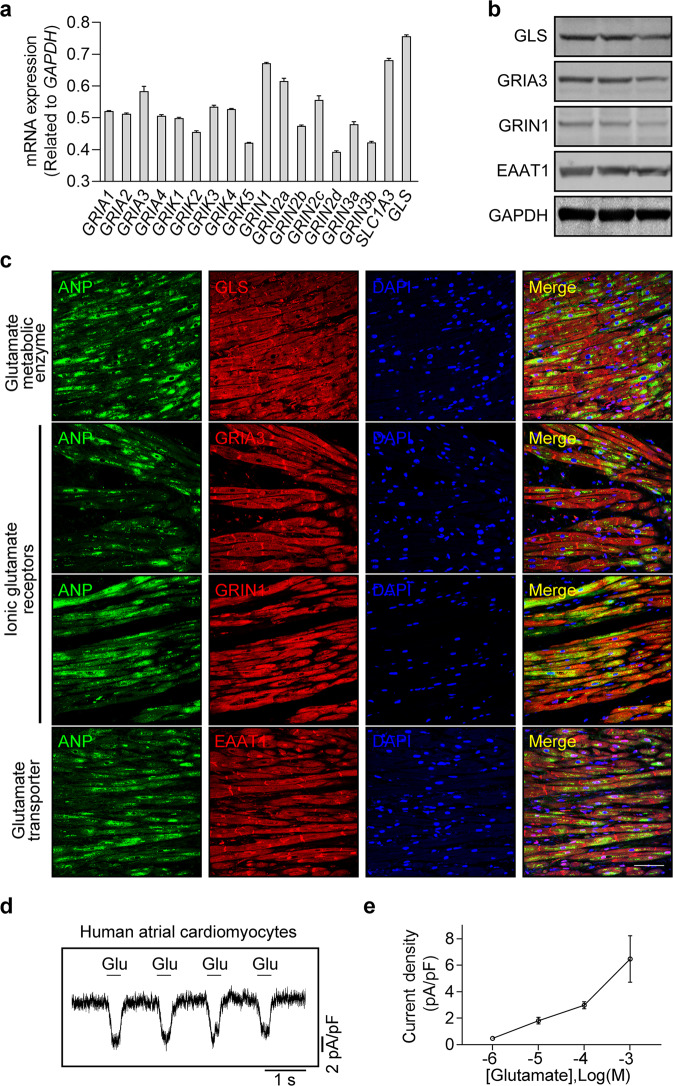
Expression and functional role of the glutamatergic transmitter system in human atrial cardiomyocytes. **a** qPCR analysis of the core elements of the glutamatergic transmitter system in human atrial cardiomyocytes illustrating that *GLS*, *GRIA3*, *GRIN1* and *SLC1A3* were highly expressed in human atrial cardiomyocytes at mRNA level. **b** Western blot analysis of the core elements of the glutamatergic transmitter system illustrating that GLS, GRIA3, GRIN1 and EAAT1 are expressed in human atrial cardiomyocytes at protein level. **c** Immunofluorescence images of the core elements of the glutamatergic transmitter system in human atrial samples showing the spatial location of GLS, GRIA3, GRIN1, and EAAT1 in the human atrial cardiomyocytes. Scale bar, 50 μm. **d** Representative patch-clamp recordings of whole-cell currents elicited by the application of 1 mM glutamate in human atrial cardiomyocytes. The holding potential was set at −60 mV. **e** Pooled data of the glutamate-elicited currents induced by different concentrations of glutamate in isolated human atrial cardiomyocytes. *n* = 6.

Next, we sought to examine whether the glutamatergic transmitter system is also present in human atria, as this is not only biologically important, but will also provide novel insights into the clinical treatment of atrial arrhythmias such as AF. We therefore performed qPCR, western blot, and immunofluorescence analyses to examine the mRNA and protein expression levels of *GLS*, *GRIA3*, *GRIN1,* and *SLC1A3* in human atrial samples. As shown in Fig. 9a–c, similar to the results in rat atrial cardiomyocytes, we found that GLS, GRIA3, GRIN1, and EAAT1 were highly expressed in human atrial cardiomyocytes, implying that the glutamatergic transmitter system may also exist in human atrial cardiomyocytes. Subsequently, we isolated the human atrial cardiomyocytes and immediately treated the cells with glutamate to monitor the electrophysiological changes. As shown in Fig. 9d, e, glutamate-evoked inward currents were detected in these human atrial cardiomyocytes, suggesting the presence of a functional glutamatergic transmitter system in human atrial cardiomyocytes as well.

## Discussion

In the present study, we identified an intrinsic glutamatergic transmitter system in atrial cardiomyocytes, which modulates the excitability and conductivity of the atrial cardiomyocytes. Our main findings can be summarized as follows. Firstly, by TEM and confocal microscopy analyses, we identified the enriched vesicles containing glutamate beneath the plasma membrane of atrial cardiomyocyte. Single-cell qPCR and immunofluorescence analyses showed that the key elements of glutamatergic transmitter system (i.e., the glutamate metabolic enzyme, the iGluRs, and the glutamate transporters) exist in atrial cardiomyocytes. Secondly, glutamate or other iGluR agonists evoked iGluR-gated currents in atrial cardiomyocytes. iGluR agonists and antagonists regulated the generation of action potentials in single atrial cardiomyocytes. Thirdly, iGluR inhibition by antagonists or knockdown of two iGluR-coding genes (*Gria3* and *Grin1*) decreased the cardiomyocyte excitability and slowed down the conduction velocity of electrical excitation in atrial cardiomyocytes. Fourthly, iGluR antagonists not only prevented but also terminated AF in isolated rat heart.

Generally, it is well known that as an excitatory neurotransmitter of the CNS, glutamate regulates the excitability and conductivity of neurons through the glutamatergic neurotransmitter system.^17^ However, to the best of our knowledge, the existence of an intrinsic glutamatergic transmitter system in cardiomyocytes has not been reported yet. The discovery of the functional transmitter system in atrial cardiomyocyte goes beyond the conventional understanding, which may make the neuron-like function one of the basic properties of the atrial cardiomyocytes. Furthermore, our data showed that the intrinsic glutamatergic transmitter system is likely to be functional in human atrial cardiomyocytes as well, suggesting that this system has potential clinical significance.

The generation of propagative action potentials in cardiomyocytes is a prerequisite for the conduction of electrical excitation in heart. Thus, we first studied the effect of the glutamatergic transmitter system on the action potentials of atrial cardiomyocytes. Our data show that iGluR agonists can not only generate the iGluR-gated currents, but also facilitate the evoking of action potentials in atrial cardiomyocytes, suggesting that the glutamatergic transmitter system can regulate the excitability of atrial cardiomyocytes. It has been well documented that adrenergic and cholinergic nerves regulate the bioelectrical activity of the heart, but they are foreign innervations of atrial cardiomyocytes. The adrenergic and cholinergic innervations differ substantially from the intrinsic regulation of atrial cardiomyocyte excitability mediated by the intrinsic glutamatergic transmitter system.

Over the past 100 years, the sinus node, atrioventricular node, and the His–Purkinje system that compose the main part of the cardiac conduction system were successively discovered.^18^ The property of gap junction channels dictates the role of the His–Purkinje system as the executor of rapid conduction of electrical impulses in ventricular myocardium,^19^ ensuring the synchronous activation of the ventricular myocardium to achieve the most efficient pumping activity.^20^ However, the His–Purkinje system only exists in ventricular myocardium, and there is no evidence of any similar system in atrial myocardium so far.^21^ Therefore, we hypothesized that there might be other electrical impulses-regulating entities in the atrial myocardium that are different from the His–Purkinje system in ventricular myocardium. Our data showed that the iGluR antagonists largely attenuated the conduction velocity of electrical impulses in both atrial myocardium and cultured atrial cardiomyocyte monolayers. Moreover, knockdown of the two atrial preferential iGluR subtypes also significantly slowed down the electrical conduction velocity in cultured atrial cardiomyocytes monolayers. These results suggested that glutamatergic transmitter system assists in electrical excitation conduction between atrial cardiomyocytes.

AF is the most common persistent cardiac arrhythmia.^22^ The pathogenesis and intervention of AF have been a major challenge in cardiology.^23,24^ One of the important pathophysiological characteristics of AF is the abnormal electrical excitability and conductivity of atrial cardiomyocytes.^25^ After confirming that the glutamatergic transmitter system participates in regulating the electrical excitability and the conductivity of the atrial cardiomyocytes, we explored whether this system can be targeted to prevent or alleviate AF. As shown in our results, iGluR antagonists can not only effectively prevent the occurrence of AF, but also effectively stop its progression, which presents a new concept for the intervention of AF. Actually, some of previous studies have implied that glutamate and its receptors participate in regulating cardiac electrophysiology or arrhythmias,^26–30^ which are consistent with our data. However, this is the first time to report that glutamate acts as a transmitter to mediate the excitation and transmission of excitation in atrial cardiomyocytes.

In summary, our findings suggest that atrial cardiomyocytes have an intact glutamatergic transmitter system, which can modulate their excitability and conductivity. Of clinical interest, iGluR antagonists can not only effectively prevent the occurrence of AF, but also significantly interrupt its progression. Our study reveals novel functional properties of atrial cardiomyocytes, and suggests that the plasticity of the excitability and conductivity of the atrial cardiomyocytes is at least partially regulated by an intrinsic glutamatergic transmitter system, providing a potential target for groundbreaking intervention of atrial arrhythmias.

## Materials and methods

### Animals

This study is conformed to the Guide for the Care and Use of Laboratory Animals made by the U.S. National Institutes of Health. All the animal experiments were approved by the Animal Care and Use Committee at Tongji University, School of Medicine.

### Ethics and acquirement of human atrial samples

The acquisition of human atrial samples was approved by the ethics committee of Zhongshan Hospital, Fudan University and was in compliance with the Declaration of Helsinki. Human atrial samples were obtained during surgery at Zhongshan Hospital, Fudan University. The atrial myocardial specimens were collected from the adult patients with normal sinus rhythm who underwent heart valve surgery. All participants have provided written informed consent.

### Electron microscopy

Fresh atrial myocardium was sectioned into 1 mm tissue blocks, and immediately immersed in ice-cold 2.5% glutaraldehyde (buffered with 0.1 M phosphate buffer, pH 7.4). The samples were fixed for at least 1 h, at 4 °C. The fixed atrial tissues were repeatedly washed in phosphate buffer and post-fixed in 1% osmium tetroxide for 1 h. Fixed specimens were dehydrated in increasing concentrations of ethanol (10%, 50%, 70%, and 90%), and then embedded in 100% epoxy resin and left to polymerize at 55 °C in 5% CO_2_ for 36 h. The resin blocks were then sectioned with an ultramicrotome. The ultrathin sections were placed on the grids, stained with uranyl acetate, and lead citrate solution for TEM observation (JOEL TEM1230, Japan).

### Isolation of rat and human atrial cardiomyocytes

Single atrial cardiomyocytes were obtained from the right atrium of adult male Sprague–Dawley rats (6–8 weeks) by enzymatic bulk digestion as previously described with minor modification.^31^ Briefly, the rat was anesthetized with pentobarbital (25 mg/kg) intraperitoneally, the chest was opened to expose the heart. The descending aorta was cut, and the heart was immediately washed by injection of 15 mL cold EDTA-Tyrode’s solution: 140 mM NaCl, 5.4 mM KCl, 1.2 mM KH_2_PO_4_, 1.8 mM CaCl_2_, 1.0 mM MgCl_2_, 5.5 mM glucose, 5 mM HEPES, and 5 mM EDTA (pH was adjusted to 7.4 with NaOH) into the right ventricle. The heart was dissected and transferred to a 60-mm dish containing fresh EDTA buffer to remove fat and connective tissues. The atrial tissues were cut into small pieces and washed twice in low-Ca^2+^ solution containing: 140 mM NaCl, 5.4 mM KCl, 1.2 mM KH_2_PO_4_, 0.2 mM CaCl_2_, 18.5 mM glucose, 50 mM taurine, and 1.0% BSA (pH was adjusted to 6.9 with NaOH) and then digested for 10–15 min in low-Ca^2+^ solution containing collagenase type II (0.8 mg/mL; Worthington, USA) with gentle agitation. The supernatant was transferred to modified Kraftbruhe (KB) solution containing: 100 mM potassium glutamate, 10 mM potassium aspartate, 25 mM KCl, 10 mM KH_2_PO_4_, 2 mM MgSO_4_, 20 mM taurine, 5 mM creatine, 0.5 mM EGTA, 20 mM glucose, 5 mM HEPES and 1.0% BSA (pH was adjusted to 7.2 with KOH) to terminate the digestion. The cell suspensions were filtered with a 100-μm filter and centrifuged at 100× *g* for 1 min to pellet the atrial cardiomyocytes. The supernatant was removed and pelleted atrial cardiomyocytes were resuspended in 4 mL (depending on the yield) KB solution. For electrophysiological experiments, gradient recalcification for the acute isolated cells was performed by using 1 mM Ca^2+^ solution per 30 min to a final concentration of 1.8 mM.

The isolation of human atrial cardiomyocytes was performed as described previously.^32^ The human atrial specimens were first placed into ice-cold Ca^2+^-free Tyrode’s solution (100 mM NaCl, 10 mM KCl, 1.2 mM KH_2_PO_4_, 5 mM MgSO_4_, 50 mM taurine, 5 mM MOPS, and 20 mM glucose, pH was adjusted to 7.0 with NaOH) supplemented with 2,3-butanedione monoxime (BDM, 30 mM, Sigma, USA), chopped into small pieces, and washed 3 times for 5 min each with Ca^2+^-free Tyrode’s solution. At all steps, the solutions were oxygenated with 100% O_2_ at 35 °C. The small pieces were then transferred into Ca^2+^-free Tyrode’s solution containing 286 U/mL collagenase type I (Worthington, USA) and 3.5–5 U/mL protease type XXIV (Sigma, USA) and gently digested for 15 min, then the supernatant was discarded. The following digestions only included collagenase type I and the Ca^2+^ concentration was raised to 0.2 mM. Stirring was continued until rod-shaped striated cardiomyocytes were seen. The suspension was gently centrifuged (100× *g*), and cardiomyocytes were resuspended and stored until use in Ca^2+^ (0.5 mM)-containing Tyrode’s solution (without BDM) at room temperature.

### Immunofluorescence analysis

For the immunofluorescence analysis of rat atrial cardiomyocytes, cells were fixed with fresh 4% paraformaldehyde for 15 min and washed twice with PBS for 5 min. The fixed cells were then permeabilized in 0.1% Triton X-100 in PBS (PBST) for 10 min, blocked with 4% normal goat serum for 1 h at room temperature, and incubated with primary antibodies at 4 °C overnight. Primary antibodies used are listed as follows: anti-ANP (Santa Cruz, USA), anti-GLS (Abcam, USA), anti-GRIA3 (Abcam, USA), anti-GRIN1 (Novus, USA), anti-EAAT1 (Abcam, USA), anti-CAST (Invitrogen, USA), and anti-Glutamate (Sigma, USA). The cells were then washed with PBST and incubated with secondary antibodies conjugated with Alexa Fluor (Abcam, USA) for 1 h at room temperature. After three washes with PBST, the nuclei were stained with DAPI. Single-section or *z*-stack confocal images were acquired on a Leica SP8 confocal laser scanning microscope (Leica, USA) using 63× immersion objective lens. The 3D reconstruction and fluorescence analysis of images were performed with LAS X (Leica, USA) and ImageJ 2.0 (NIH, USA).

For the immunofluorescence staining of atrial tissue sections, the samples were fixed with 4% paraformaldehyde, paraffin-embedded, and sectioned, the tissue sections were de-paraffinized with xylene and rehydrated through a series of decreasing concentrations of ethanol. Antigen retrieval was performed by microwaving in citrate solution for 10 min; then, the slides were blocked with 10% goat serum and incubated with primary antibody at 4 °C overnight. On the next day, the slides were washed in PBST three times to remove unbound antibodies and then incubated with the appropriate fluorescence-conjugated secondary antibody in PBST with 10% goat serum for 1 h at room temperature. The slides were washed in PBST three times and then DAPI staining (Sigma, USA) was performed to visualize the nuclei.

### RNA extraction and qPCR

For single-cell RNA extraction, individual cells were manually picked under the microscope and transferred into 1.5 μL cell lysis buffer to extract total RNA. RNA was reversed to first-strand cDNA through 9 cycles using Superscript III (Invitrogen, USA) and then amplificated for 18 PCR cycles with KAPA polymerase (Kapa Biosystems, USA). cDNA quantification was performed with Qubit 3.0 (Thermo Fisher, USA). The cDNA was properly diluted and used as template for qPCR.

All the qPCR reactions were performed with the SYBR Premix Ex Taq Kit (TAKARA, Japan) on the QuantStudio 6 Flex (Applied Biosystems, USA). The qPCR data were generated with QuantStudio Real-Time PCR system software. In addition, the semi-qPCR products was analyzed by 2% agarose gel electrophoresis. Primers used are listed in Supplementary information, Table S1.

### Western blot analysis

Total proteins were extracted through a conventional method using RIPA lysis buffer (Beyotime Biotechnology, China) with protease inhibitor cocktail (Beyotime Biotechnology, China) at 4 °C. Protein concentrations were determined by BCA protein assay (Beyotime Biotechnology, China), and equal amounts of total proteins were separated by 10% SDS-PAGE (Thermo Fisher Scientific, USA), and then transferred onto PVDF membranes (Millipore, USA). Next, the membranes were blocked with 5% nonfat milk in 0.1% Tween washing buffer for 1 h at room temperature, and then incubated with primary antibodies including anti-GLS (Abcam, USA), anti-GRIA3 (Abcam, USA), anti-GRIN1 (Novus, USA), anti-EAAT1 (Abcam, USA) and anti-GAPDH (Abcam, USA) overnight at 4 °C. On the next day, the membranes were incubated with the conjugated secondary antibody (Invitrogen, USA) for 1 h at room temperature and the bands were visualized using ChemiDoc Touch Gel Imaging System (Bio-Rad, USA). The blots were quantified by densitometry using ImageJ software (NIH).

### Patch clamp

Whole-cell patch clamp was applied to record the membrane potentials and currents. Two borosilicate glass microelectrodes were used: one for electrical stimulation and recording with 2–5 MΩ resistances filled with pipette solution and the other for pulsatile administration with a tip of 10 μM inner diameter filled with the bath solution or bath solution containing different pharmacological agents. Pressure in the application pipette channels was adjusted using the PL1–100 picoinjector microinjector (Harvard Apparatus, USA). The stimulation pulses were applied via EPC-10 amplifier (HEKA, Germany) synchronized with the microinjector for the synchronous drug release. The action potential or the current was recorded in the current clamp or the voltage-clamp mode, all the data were collected through an EPC-10 amplifier with standard patch-clamp techniques at room temperature. The bath solution contained the following ingredients: 135 mM NaCl, 10 mM glucose, 5.4 mM KCl, 1.8 mM CaCl_2_, 0.3 mM Na_2_HPO_4_, 0.3 mM KH_2_PO_4_, 10 mM HEPES (pH 7.35, adjusted with NaOH). The standard pipette solution contained the following ingredients: 140 mM KCl, 10 mM EGTA, 10 mM HEPES, 5 mM glucose, 3 mM Na_2_ATP, pH was adjusted to 7.2 with KOH. The data were analyzed with Patchmaster 2.42 (HEKA, Germany) and Clampfit 10.7 (Molecular Devices, CA) software.

### Atrial conduction velocity measurements in vivo

To implant the cardiac catheter in the atria through the right jugular vein, a 5-cm incision was made on the right side of the neck, and the external jugular vein was exposed by blunt dissection. Then, 1.1F electrophysiology catheter (Millar Instruments, USA) was inserted into the right atria and the electrical signals were recorded by the 16-channels PowerLab (ADInstruments, USA). The electrocardiograms were collected and analyzed by the LabChart 7 (ADInstruments, USA) and the conduction velocity was calculated by using the equation: CV = Δs/Δt, in which Δs is the distance between the two electrodes and Δt is the time between the points with the maximum depolarization slope in electrocardiograms.

### Optical mapping

Voltage-sensitive dye-based optical mapping was performed as described previously.^33^ Briefly, adult male Sprague–Dawley rats (6–8 weeks old) were anesthetized with pentobarbital (25 mg/kg) intraperitoneally containing 120 IU of heparin. Following a midsternal incision, the heart was rapidly excised and Langendorff-perfused at 37 °C with oxygenated (95% O_2_ and 5% CO_2_) standard Tyrode’s solution. The heart was stained with the voltage-sensitive dye RH237 (30 μM, AAT, USA) for 5 min. Blebbistatin (10 μM, MCE, USA) was added to the perfusate to eliminate motion artifacts during optical recordings. The dye was excited using LED light sources centered at 550 nm. Images were captured with a high-speed 10,000 pixels camera (SciMedia, MiCAM ULTIMA, USA) at 1000 frames/s. Data analysis was performed using Brain vision 16.04 (SciMedia, USA) programs designed for the analysis of optically recorded action potentials. Local activation was determined as the time point of maximum change in fluorescence over time for each fluorescent signal in the image. The conduction velocity was calculated from the gradient of the activation maps.

### MEA recording in human iPSC-derived atrial cardiomyocytes

The human iPSC-derived atrial cardiomyocytes were obtained from the Institute of Biophysics of the Chinese Academy of Sciences (Beijing, China). After recovery, the cells were cultured in a dish precoated with vitronectin (0.01 μg/μL, Cauliscell, China) with cardiomyocyte maintenance media (Cauliscell, China) at 37 °C, under 5% CO_2_. The medium was changed every 2 days. On day 5, the cells were dissociated and resuspended in cardiac maintenance media at a density of 1 × 10^7^ cells/mL. Aliquots of 10 µL of cell suspension were plated onto CytoView MEA 24 plates (Axion BioSystems, USA) for potential recording. The MEA device automatically adjusted and controlled the environment (37 °C and 5% CO_2_) to maintain the temperature and medium pH. The data were acquired using the Maestro Pro multi-well MEA platform (Axion BioSystems, USA).

### siRNA transfection

The siRNA-mediated knockdown of *GRIA3* and *GRIN1* was performed with the lipofectamine RNAiMAX (Invitrogen, USA) following the manufacturer’s guidelines. Briefly, the cultured human iPSC-derived atrial cardiomyocytes were transfected with a final concentration of ∼20 pmol siRNA together with 3 μL lipofectamine RNAiMAX per 500 μL. The follow-up experiments were performed 72 h after transfection. All the siRNA sequences are listed in Supplementary information, Table S2.

### Langendorff heart perfusion and AF induction

The rat AF induction model was based on a previously described study.^34^ Briefly, the rat was anesthetized with pentobarbital (25 mg/kg) intraperitoneally, the chest was opened to expose the heart. Then the heart was quickly removed, and mounted in the Langendorff perfusion system perfused with oxygenated perfusion solution containing 135 mM NaCl, 10 mM glucose, 5.4 mM KCl, 1.8 mM CaCl_2_, 0.3 mM Na_2_HPO_4_, 0.3 mM KH_2_PO_4_, 10 mM HEPES (pH 7.35, adjusted with NaOH) at 37 °C. The electrocardiogram (ECG) and intracardiac recordings from the right atria (RAE) was continuously recorded from the Langendorff-perfused heart with Powerlab amplifier (Powerlab, AD Instruments, USA). A 1.1F electrophysiology catheter (Millar Instruments, USA) was inserted through the jugular vein to pace the inner wall of the right atrium. The burst pacing was generated by an external stimulator (STG-3008-FA, Multi-Channel Systems, Germany). Pacing threshold was determined by incrementally increasing the voltage until atrial capture occurred; pacing was then performed at 2× pacing threshold. To induce AF, reduced perfusion and burst pacing were utilized. After the balance of perfusion for 30 min, we reduced the coronary flow from 6 mL/min to 3 mL/min, and paced the atria with 40 Hz burst pacing for 1 min (repeated up to ten times at an interval of 1 min) to induce AF. Each rat underwent an identical pacing and programmed stimulation protocol. Successful induction of AF was defined as irregular atrial rates in RAE lasting longer than 2 min, and the induction rate was defined as the number of successfully induced hearts to the total stimulated hearts.

### Statistical analysis

Normality was tested using the Kolmogorov–Smirnov test. Two-tailed Student’s *t*-test or nonparametric test was used for comparisons between two groups. The induction rate of AF was analyzed using the *χ*^*2*^ test. Statistical significance was defined as a *P* value < 0.05. The normally distributed data are shown as means ± standard error of mean (SEM), and the non-normally distributed data are shown as medians ± interquartile range (IQR). All statistical analyses were performed with GraphPad Prism 8.

## Supplementary information

Supplementary Table S1

Supplementary Table S2

Supplementary information, Video S1

Supplementary information, Video legend
