# Supplementary material for: Identification of an endogenous glutamatergic transmitter system controlling excitability and conductivity of atrial cardiomyocytes
Source: Cell Res. 2021 Apr 6;31(9):951–64. doi: 10.1038/s41422-021-00499-5 (PMC8410866; doi:10.1038/s41422-021-00499-5)
Supplement: Supplementary file 1 — Supplementary Table S1 [file 41422_2021_499_MOESM1_ESM.pdf]

Supplementary Materials For

Title: Identification of an endogenous glutamatergic transmitter system controlling excitability and conductivity of atrial cardiomyocytes.

This file includes:

Supplementary information, Tables S1

Table S1. List of primers used for quantitative PCR (qPCR).

Gene	Species	Forward Primer	Reverse Primer
Nppa	Rat	CAACACAGATCTGATGGATTTCA	CCTCATCTTCTACCGGCATC
Gls	Rat	GAATAAGATGGCTGGTAATGAATATG	ACACTTGCTGACTCACACG
Gria1	Rat	AGGCTGGTGGTGGTTGAC	ACATTGGCTCCGCTCTCC
Gria2	Rat	CAACACTGGAAGAGAAAGAATACC	TCACTTGGACAGCATCATACG
Gria3	Rat	TTATACAACACCAACCAGAACAC	ATAGAATCCAAAGATAGCATACACC
Gria4	Rat	TTGTGATAGATTGTGAGATAGAGAG	TTGCGATGATATAATGGTAGCC
Grik1	Rat	CGGACTATGCGGCTATCAG	GAGGTTTGGCGTCTTTATTCG
Grik2	Rat	CTGATGCTGCTCTGATGTATG	CTGCCTGTGAGACCTTCC
Grik3	Rat	CGAGGAGGAGAACAAGGAG	AACACGGACAGGACTAAGC
Grik4	Rat	GCTTCCTGCTTGGCTCTTG	CGGTTGATGCGGTTCTTGG
Grik5	Rat	GCATCTGTCAGCCTGTATCC	GCGGTGATGGTGAAGGC
Grin1	Rat	CAGTGAGGAGGAGGAGGAAG	AAGTTGGCAGTGTAGGAAGC
Grin2a	Rat	CAAGACAACAGTGGACAACAG	AGCAGGATGACAGAAGAATGG
Grin2b	Rat	TTATCTCTGTGTCTTATGATGAATGG	GCAGCAGTGGTGATGATGG
Grin2c	Rat	GGTGATGATGTTCGTGATGTG	GGTGAGGTTCTGGTTGTAGC
Grin2d	Rat	ACAACCAGCCGAGAGTG	GCGATGCCATAACCAGTG
Grin3a	Rat	AAACGTGTGGAGAAGAGCAGAT	TGGACCTAGGAAACAGAGACAAT
Grin3b	Rat	TGGCACTGTCTTCTCTTACTC	CACTTGGGCGTCTTACTGG
Grina	Rat	GAGCCCAGAAGAATATGTGTTTG	GGTGAGAATGTATAGGAAGATGTTG
Grid1	Rat	TGGACTTCAGCAAGCGATAC	CAACACGAATATGAGCACACC
Grm1	Rat	CCGAAGAAGATGAATTGGAAGAG	CAGAATGACAGAGGCGTAGG
Grm2	Rat	AATCTCCTGCGGCTGTTC	CCTGTCTCACCATAGTCACC
Grm3	Rat	CCAGAGAAGCGGGAAACAG	GAATGCCAACCAGATGATGC
Grm4	Rat	CCAGGACCAACGGACAC	CCAGCAGGCAGATGAGG
Grm5	Rat	CAATCTATTCTATGGCTTATGGACTC	GCTTCATCGCATCACAGAGG
Grm6	Rat	ACTACGAGGAGCAGAGGAC	AAGCCAGCCAGATAATACAGG
Grm7	Rat	TTGTGATTGATGCTGTCTATGC	TTCTTGCCGCCTGCTTG
Grm8	Rat	TGGATTGGCTCTGATAGTTGG	ATTGTCACCGCTCCTTCTG
Slc1a1	Rat	TCATAGTCGTGCGGAAGAAC	AGCGGAATGTAACTGGAAGG
Slc1a2	Rat	ACGACACGAAGAACCACAG	CCGCCAGAGTTACCTTGC
Slc1a3	Rat	GCTTCTGATGCGGATGCTG	TGGATGATGATGACAATGATTATGC
Slc1a4	Rat	TGTTGCTCTGGCGTTCATC	TTGGAGACTGGAGGAGGTTC
Slc1a5	Rat	CGGCAAGATTGTGGAGATG	AAGTAGATGAGAGGCAGAACC
Slc1a6	Rat	TTCTCCTCCATCCTCAGCAG	GGCACAGTCTCCTCAAAGC
Slc1a7	Rat	CTGTCTGTGCTGTCTGTCATC	GCGGCTGGAGGTCTTGG
GLS	Human	ACCTTTGAAATATGCCATTGCTG	GTTTGTTGAATCTTAGTCCACTCG
GRIA1	Human	TGCTCAAGAAGAACGCCAATC	TGACAATCTCCAGACGGTAGG
GRIA2	Human	AGAGTGCGGAAGTCCAAAGG	GGTGTTGCGATGCCATAGC
GRIA3	Human	TTACATTGGAGTCAGCGTAGTTC	CGAGGTTCTTCATTGTTGTCTTC
GRIA4	Human	GGTGTCAGCGTGGTCTTATTC	GGTCCTTCCTTTCCGTCCTC
GRID1	Human	TCCTCCTACACAGCCAAC	TACTCATATACAGCAGAATCCC
GRIK1	Human	TCTTGGTGTGGCTGCTCTC	GGTCTGTATGTGTGGAACTTCG

GRIK2	Human	AGGATAGTGGGAGGCATTTGG	TAAATCATCAGCAGAGTCAATAGGG
GRIK3	Human	TATCGTGTCCGTGTGCTACC	ACAATTCGTCCAGTTAATCCTTCC
GRIK4	Human	CCAGAGCCTCAACCAGTCC	CGCAGTCACCACAGCATAG
GRIK5	Human	CGCCAACGCCTCCATCTC	GTCCTCCACAATACCGTCCAG
GRIN1	Human	GTGCCTCAGTGTTGCTACG	TCGCCCATCATCCCATTCC
GRIN2A	Human	GTTTGTGGGTGATGGTGAGATG	AGATGAAGGTGATGAGGCTAAGG
GRIN2B	Human	CACCACTGCTGCTTCTGAC	CCCTTTCCCACTTCCTCTCC
GRIN2C	Human	GGGACGGAGAGACACAGAAAC	CCAGGCGAAGACCAGCAG
GRIN2D	Human	GGCTTCCACCGCTACTACG	CTCGGCTGGCTCCTTGTC
GRIN3A	Human	TCATCAACATCACCGCTAACC	GTCCTCAGTTCCTCCACATTC
GRIN3B	Human	CGAGCACGCCTTCTTCCG	CCTCTGCCGTCTCCTCCTTC
GRINA	Human	CTTCATCCGCAAGGTGTTCC	AGACAGCATAGGAGACATAGTAGG
GRM1	Human	CTCTTCCGTGGCTCTGG	GCAATGGGCTTCTTAGTCC
GRM2	Human	CTGGTGTTATTGGCGGTTCC	GCGGGCAAAGTAGTCATAGC
GRM3	Human	GCAGGAGAGCATCATCAAGG	GCGGTGGTTGTTGTAGGG
GRM4	Human	CGAGAAGGATGGCACAGAG	TGAAGCACCGATGACACC
GRM5	Human	GCTGGCATTCGGCTGTG	GCGGAAGGAAGAGGAGGAG
GRM6	Human	CCAGGAGAGTTCAGCAAGG	TGAGCCGACCCACAGG
GRM7	Human	TTCTTGGGCTTGGGTATGTG	ATGTTGGGCTTATGAGTCTGG
GRM8	Human	CTCCTTTCCAACATCACTCTG	ATCTCCATTAGCACACTTCAC
SLC1A3	Human	GGACCTCTTCAAGTTCTGCCA	GGCAGCCAAAGCCTCATAGA
GAPDH	Human	AGCCACATCGCTCAGACAC	GCCCAATACGACCAAATCC
