# Supplementary material for: Identification of an endogenous glutamatergic transmitter system controlling excitability and conductivity of atrial cardiomyocytes
Source: Cell Res. 2021 Apr 6;31(9):951–64. doi: 10.1038/s41422-021-00499-5 (PMC8410866; doi:10.1038/s41422-021-00499-5)
Supplement: Supplementary file 2 — Supplementary Table S2 [file 41422_2021_499_MOESM2_ESM.pdf]

Supplementary Materials For

Title: Identification of an endogenous glutamatergic transmitter system controlling excitability and conductivity of atrial cardiomyocytes.

This file includes:

Supplementary information, Table S2

Table S2. siRNAs sequences list.

Gene	Species	Sequences (5'to 3')
Gria3	Human	GGCGCAUCAUUGAAGAAAUTT
		AUUUCUUCAAUGAUGCGCCTT
Grin1	Human	GGCAUAUGGAGAAGCACAATT
		UUGUGCUUCUCCAUAUGCCTT
