# Supplementary material for: Identification of an endogenous glutamatergic transmitter system controlling excitability and conductivity of atrial cardiomyocytes
Source: Cell Res. 2021 Apr 6;31(9):951–64. doi: 10.1038/s41422-021-00499-5 (PMC8410866; doi:10.1038/s41422-021-00499-5)
Supplement: Supplementary file 4 — Supplementary information, Video legend [file 41422_2021_499_MOESM4_ESM.pdf]

Supplementary Materials For

Title: Identification of an endogenous glutamatergic transmitter system controlling excitability and conductivity of atrial cardiomyocytes.

This file includes:

Supplementary information, Legend for Video S1

Supplementary Video Legend

Video S1. 3D reconstruction of the atrial cardiomyocyte in Fig. 1c - representing the spatial relationship between glutamate (red) and CAST (green).
